# Thidiazuron Triggers Morphogenesis in *Rosa canina* L. Protocorm-Like Bodies by Changing Incipient Cell Fate

**DOI:** 10.3389/fpls.2016.00557

**Published:** 2016-05-04

**Authors:** Yaping Kou, Cunquan Yuan, Qingcui Zhao, Guoqin Liu, Jing Nie, Zhimin Ma, Chenxia Cheng, Jaime A. Teixeira da Silva, Liangjun Zhao

**Affiliations:** ^1^Beijing Key Laboratory of Development and Quality Control of Ornamental Crops, Department of Ornamental Horticulture and Landscape Architecture, China Agricultural UniversityBeijing, China; ^2^National Engineering Research Center for Floriculture, Beijing Forestry UniversityBeijing, China; ^3^Independent ResearcherMiki-cho, Japan

**Keywords:** auxin, cytokinin, PLB, rhizoids, *Rosa canina*, stem cell, TDZ

## Abstract

Thidiazuron (*N*-phenyl-*N*′-1,2,3-thiadiazol-5-ylurea; TDZ) is an artificial plant growth regulator that is widely used in plant tissue culture. Protocorm-like bodies (PLBs) induced by TDZ serve as an efficient and rapid *in vitro* regeneration system in *Rosa* species. Despite this, the mechanism of PLB induction remains relatively unclear. TDZ, which can affect the level of endogenous auxins and cytokinins, converts the cell fate of rhizoid tips and triggers PLB formation and plantlet regeneration in *Rosa canina* L. In callus-rhizoids, which are rhizoids that co-develop from callus, auxin and a Z-type cytokinin accumulated after applying TDZ, and transcription of the auxin transporter gene *RcPIN1* was repressed. The expression of *RcARF4*, *RcRR1*, *RcCKX2*, *RcCKX3*, and *RcLOG1* increased in callus-rhizoids and rhizoid tips while the transcription of an auxin response factor (*RcARF1*) and auxin transport proteins (*RcPIN2*, *RcPIN3*) decreased in callus-rhizoids but increased in rhizoid tips. *In situ* hybridization of rhizoids showed that *RcWUS* and *RcSERK1* were highly expressed in columella cells and root stem cells resulting in the conversion of cell fate into shoot apical meristems or embryogenic callus. In addition, transgenic *XVE::RcWUS* lines showed repressed *RcWUS* overexpression while *RcWUS* had no effect on PLB morphogenesis. Furthermore, higher expression of the root stem cell marker *RcWOX5* and root stem cell maintenance regulator genes *RcPLT1* and *RcPLT2* indicated the presence of a dedifferentiation developmental pathway in the stem cell niche of rhizoids. Viewed together, our results indicate that different cells in rhizoid tips acquired regeneration competence after induction by TDZ. A novel developmental pathway containing different cell types during PLB formation was identified by analyzing the endogenous auxin and cytokinin content. This study also provides a deeper understanding of the mechanisms underlying *in vitro* regeneration in *Rosa*.

## Introduction

*Rosa* species are widely cultivated around the world because of their ornamental and high economic value (Debener and Linde, 2009). Over the past decade, genetic engineering and genomic approaches have been used to improve several traits in the genus *Rosa*, including disease resistance, recurrent flowering, scent production, flower color, and flower architecture (Tanaka et al., 2005; Zlesak, 2009). Several regeneration pathways have been explored and used for rose transformation (Firoozabady et al., 1994; Hsia and Korban, 1996; Kim et al., 2003, 2004; Chen et al., 2006, 2010; Pati et al., 2006; Randoux et al., 2014). However, regeneration systems of *Rosa* plants *in* *vitro* continue to be inefficient and time-consuming, seriously limiting the application of transgenic engineering to this ornamental. Tian et al. (2008) established an efficient and rapid regeneration system using protocorm-like bodies (PLBs) that formed in response to thidiazuron (*N*-phenyl-*N*′-1,2,3-thiadiazol-5-ylurea; TDZ) (Tian et al., 2008). In this PLB regeneration system, leaves excised from 6-week-old shoots were first used to induce callus and rhizoids (a root-like structure that has no root cap as in a normal root, but instead only possessing a root apical meristem (RAM) and root stem niche) *in vitro* in the dark on Murashige and Skoog (MS) (Murashige and Skoog, 1962) basal medium supplement with 1.5 mg/L 2,4-D. In that study, PLBs that formed on the tips of rhizoids turned green and were bulbous after subculture on PLB induction medium (PIM), which consists of half-strength (micro- and macronutrients) MS basal medium (1/2 MS) with 20 mg/L TDZ and 2% sucrose. PLBs, which are developmental structures generally associated with structures similar to seed-derived protocorms in orchids (Teixeira da Silva, 2014), have served as a practical approach for the *in vitro* regeneration of nine *Rosa* species (Guo, 2009). In a downstream application, the *GUS* gene was successfully transformed into *Rosa canina* L. PLBs (Bi et al., 2012). Histochemical observations showed that PLBs were derived from parenchyma cells adjacent to the epidermis of rhizoid tip cells (Liu et al., 2014). Other than this, little is known about how TDZ triggers PLB formation, especially the changes in the endogenous content of auxins and cytokinins (CKs), or in the expression of related genes.

TDZ, which is a synthetically derived urea displaying primarily CK-like activity, is the only plant growth regulator (PGR) that has been used to date to induce PLBs in *R. canina*. TDZ is used more generally in plant tissue culture for inducing shoots and somatic embryos (Li et al., 2002; Feyissa et al., 2005), protoplasts (Kim et al., 2003), and flowers (Chang and Chang, 2003) *in vitro*. TDZ can be used as a substitute for both auxin and CK (Thomas and Katterman, 1986; Visser et al., 1992; Casanova et al., 2004). However, there are few reports about the biochemical or genetic mechanisms underlying how cells acquire regeneration competence following induction by TDZ.

Plant regeneration involves successive developmental events in response to environmental cues (Fehér, 2015). The acquisition of regeneration competence is easily achieved in plant tissue culture by adjusting the exogenous levels of PGRs (Che et al., 2007). Indole-3-acetic acid (IAA) is a major form of auxin in plants (Brunoud et al., 2012). Auxin concentration gradients play a critical role in plant development both *in vivo* and *in vitro*, and auxin polarization and redirection through auxin transporter protein determine tissue morphogenesis, such as lateral organ initiation and definition of the apicobasal embryonic axis (Robert et al., 2013; Wabnik et al., 2013). Members of the *PIN-FORMED* (*PIN*) gene family encode components of the auxin efflux machinery, including *PIN-FORMED1* (*PIN1*), *PIN-FORMED2* (*PIN2*), *PIN-FORMED3* (*PIN3*), *PIN-FORMED4* (*PIN4*), and *PIN-FORMED7* (*PIN7*), which function singly or in combination to direct auxin flux and reflux in *Arabidopsis* roots (Blilou et al., 2005; Adamowski and Friml, 2015). PIN1 appears to be the primary mediator of IAA movement through vascular tissues and is essential for acropetal transport (to the root tip) of auxin in root tissue (Galweiler et al., 1998; Blilou et al., 2005). PIN2 is notable for its polarized localization and root gravitropic response (Müller et al., 1998) and also determines the redistribution of auxin in the basipetal (out of the root tip) transport stream through cortical and epidermal cells in root tips (Benkova et al., 2003; Blilou et al., 2005). PIN3, which appears to function in the lateral redistribution of auxin, and is involved in phototropic and gravitropic growth, is localization in the cells of the shoot endodermis, gravi-responding root columella and pericycle cells (Friml et al., 2002a,b). Auxin response factors (ARFs), together with the TIR1/AFB F-box and Aux/IAA proteins, constitute the auxin signal transduction pathway. The *ARF* gene family plays an important role in regulating the transcription of auxin stimulation during plant development (Tan et al., 2007; Chapman and Estelle, 2009; Guilfoyle and Hagen, 2012). There are 23 genes coding for ARF proteins in *Arabidopsis* and which serve as B3-type transcription factors (Guilfoyle and Hagen, 2007). ARF proteins can be divided into activators and repressors of auxin signaling based on their activity in protoplasts: ARFs 5, 6, 7, 8, and 19 act as activators while other ARFs act as repressors (Guilfoyle and Hagen, 2012).

Cytokinins also participate in many aspects of plant development (Werner and Schmülling, 2009). The ribosides isopentenyladenosine (iPA) and zeatin riboside (ZR) are the translocated forms of the Z- and iP-type of CK, respectively (Takei et al., 2001). CK signaling is mediated by a two-component signaling pathway, which contains the CK receptor histidine kinases (HKs), histidine phosphotransferases (HPs), and response regulators (RRs) (Hwang and Sheen, 2001; Hwang et al., 2012). In *Arabidopsis*, *ARR1* is considered to be a marker of CK signaling because of its immediate response to CK (Sakai et al., 2001) and has been reported to be critical in many processes during plant development (Jones et al., 2010). The AHK3/ARR1 two-component signaling pathway leads to decreased expression of *PIN1*, *PIN3*, and *PIN7* and impairs cell differentiation (Dello Ioio et al., 2008). To metabolize CKs in plants, iP- and Z-type CKs are released from their nucleotide precursor formed by CK nucleoside 5′-monophosphate phosphoribohydrolase *LONELY GUY* (*LOG1*) and become active (Kurakawa et al., 2007). Wong et al. (2013) showed that the expression of *GmLOG1* in the axillary shoot meristem was earlier than that of *GmWUS* or *GmCLV3* during the initial stage of axillary shoot meristem development in soybean (*Glycine max* L.), which indicates that CKs act as one of the earliest signals in initiating and specifying the shoot stem cell population. Tamar et al. (2013) noted that ectopic expression of the *LOG1* gene in tomato (*Lycopersicon esculentum* Mill.) induced aerial minitubers from tomato shoot buds. CK oxidase/dehydrogenase (CKX) are enzymes that catalyze the breakdown of all CKs in a single step (Werner et al., 2006). The CK-degrading enzyme *CYTOKININ OXIDASE 3* (*CKX3*) is expressed in the organizing center of the shoot apical meristem (SAM) (Yadav et al., 2009; Bartrina et al., 2011), and *ckx3-ckx5* double mutants displayed increased *WUS* expression and larger SAMs (Bartrina et al., 2011).

Many functional genes have been identified and confirmed to be determining factors in plant regeneration. For example, *WUSCHEL RELATED HOMEOBOX 5* (*WOX5*) is specifically expressed in the quiescent center (QC) and is considered to be a marker of the root stem cell niche (Sarkar et al., 2007). *PLETHORA 1* (*PLT1*) and *PLETHORA 2* (*PLT2*) are master regulators involved in the maintenance of basal/root fate (Aida et al., 2004; Galinha et al., 2007). Sugimoto et al. (2010) showed that *Arabidopsis* could regenerate shoots from multiple tissues, but all indirectly through a callus pathway, and that the development of callus shared the same traits as in lateral root development. In *Arabidopsis*, the root stem cell regulators *PLT1* and *PLT2* must be activated to establish the competence of shoot regeneration progenitor cells (Kareem et al., 2015). *WUSCHEL* (*WUS*) is specifically expressed in the center of SAMs, and is considered to be a marker of SAM identity. *WUS*, together with *CLAVATA 1/3* (*CLV1/3*), maintains the SAM stem cell niche through a feedback pathway in *Arabidopsis* (Schoof et al., 2000). *WUS* is also considered to induce shoot stem cell activity in roots (Gallois et al., 2004) and to convert RAMs to SAMs, depending on the exogenous PGR applied *in vitro* (Chatfield et al., 2013). *SOMATIC EMBRYOGENESIS RECEPTOR KINASE 1* (*SERK1*), which is involved in the acquisition of embryogenic competence in plant tissue culture, is strongly expressed during early stages of zygotic and somatic embryogenesis in *Arabidopsis* (Hecht et al., 2001; Zhang et al., 2014), and has a broader role in *Medicago truncatula* Gaertn. organogenesis (Nolan et al., 2003).

In this study, using the *R. canina* PLB model developmental system, the main aim was to assess how rhizoid cells attain regeneration competence following induction by TDZ. Furthermore, it was our sub-objective to assess the content of endogenous auxin and CKs in developing PLBs. Finally, another sub-objective was to observe the transcription levels of genes related to auxin, CKs, the RAM and the SAM. In doing so, we hoped to better understand the molecular inductive function of TDZ during PLB formation in *R. canina* and thus provide a better understanding of the mechanistic basis of this unique *in vitro* developmental event in *Rosa* species.

## Materials and Methods

### Plant Material and *In Vitro* Regeneration

Callus-rhizoids (i.e., rhizoids that co-develop from callus) derived from 6-week-old leaves were transferred onto PIM, which was 1/2 MS supplemented with 20 mg/L TDZ based on a previously described protocol (Tian et al., 2008). After culture for 0, 1, 2, 3, 4, or 5 days on PIM, callus-rhizoids and the terminal 1 cm of rhizoid tips were collected and stored at -80°C for later use. Cultures were maintained at 25 ± 2°C under a 16-h photoperiod with a light intensity of 25 μmol m^-2^ s^-1^ provided by cool-white fluorescent tubes.

Three *XVE::RcWUS* transgenic plant lines (#5, #8, #9) were used to assess the percentage of PLBs that formed during the early stage (the first 10 days on PIM) of PLB formation.

### Quantification of IAA, ZR, and iPA Using ELISA

Fresh callus (0.2 g) that included rhizoids (i.e., rhizoids-callus) obtained from PIM was ground into a fine powder with liquid nitrogen and dissolved in 2.0 mL of phosphate-buffered saline (PBS) containing 0.1% (v/v) Tween-20 and 0.1% (w/v) gelatin (pH 7.5) to quantify the amount of free IAA, ZR, and iPA, using an enzyme-linked immunosorbent assay (ELISA) (Yang et al., 2001). Mouse monoclonal antibodies against free IAA, ZR and iPA were produced at the Center of Crop Chemical Control, China Agricultural University, China (Weiler et al., 1981; Wang et al., 2012). ELISA data was calculated as described previously (Weiler et al., 1981).

### Treatment of Callus-Rhizoids with Exogenous Factors

1-Naphthaleneacetic acid (NAA), *N*-1-naphthylphthalamic acid (NPA, an inhibitor of auxin transport) and kinetin (KT; 6-furfuryl aminopurine) were used to assess the effect of TDZ on PLB formation. The ratio of PLB formation [(number of PLBs/total number of rhizoids per callus-rhizoid) × 100] was calculated after each treatment. One callus-rhizoid indicates a single unit of callus-rhizoids derived from one leaf (**Figures 2B,C**). For auxin treatment, leaves with callus-rhizoids were cultured on PIM containing 0.01, 0.1, or 1.0 mg/L NAA. For the NPA treatments, leaves with callus-rhizoids were cultured on PIM supplement with 10 μL NPA while for the KT treatment, leaves with callus-rhizoids were cultured on PIM containing 0.01, 0.1, or 1.0 mg/L KT. Twenty callus-rhizoids were used for each treatment, and each experiment was performed five times. After culture for 9 days, the ratio of PLB formation in each treatment was calculated.

### Quantitative Real-Time PCR

Total RNA was extracted from callus-rhizoids or rhizoids with a Huayueyang RNA extraction kit (Huayueyang, ZH120, Beijing, China). Two μL of RNA was used to synthesize cDNA in a reaction volume of 20 μL using a FastQuant RT Kit (Tiangen, KR106, Beijing, China), which contains gDNase and a random primer. cDNA was then used for quantitative real-time PCR (qRT-PCR) analysis on an ABI 7500 Real-Time PCR System (Applied Biosystems, Foster City, CA, USA) using a KAPA^TM^ SYBR^®^ FAST qPCR kit (KapaBiosystems, Woburn, MA, USA) according to the manufacturer’s instructions. The PCR protocol was: denaturation at 95°C for 30 s, 40 cycles at 95°C for 5 s for product amplification and a final extension at 60°C for 30 s. The gene-specific primers used for qRT-PCR are listed in **Supplementary Table S1**. Gene expression levels were normalized to *18S* and *UBIQUTIN2* transcripts using the comparative ΔΔCt method (Livak and Schmittgen, 2001).

### RNA *In Situ* Hybridization

Intact rhizoids approximately 1 cm long were excised from callus-rhizoids after culture on PIM for 0, 3, 5, or 9 days. Rhizoid tips (∼1 cm long) were fixed in FAA (formaldehyde, acetic acid, 50% ethanol 5:5:90, v/v/v) for a minimum of 24 h. After dehydration through an alcohol-xylol series, rhizoid tips were embedded in paraffin (Sigma–Aldrich, Mannheim, Germany) with a 58°C melting point (Liu et al., 2014). Serial longitudinal sections (10 μm thick) of the rhizoids made with a microtome (Leica RM2235, Solms, Germany) were used for *in situ* hybridization in which fragments of *RcWUS*, *RcPLT1*, or *RcSERK1* were used to generate sense and antisense RNA probes by PCR amplification using a DIG RNA Labeling Kit, according to the manufacturer’s instructions (Roche, Mannheim, Germany). PCR conditions were as follows: 95°C for 30 s, 40 cycles at 95°C for 5 s, and 58°C for 30 s. The gene-specific primer sequences are listed in **Supplementary Table S2**. All procedures were performed at Prof. Zheng Meng’s Lab (Institute of Botany, Chinese Academy of Sciences). The slides were left in a sealed and humid box in a dark drawer to prevent light contamination for 18 h, and then stained sections were examined and photographed under a BX-50 light microscope (Leica DMRE, Solms, Germany).

## Results

### Endogenous Auxin and CK Levels Were Increased by TDZ

**FIGURE 1:**
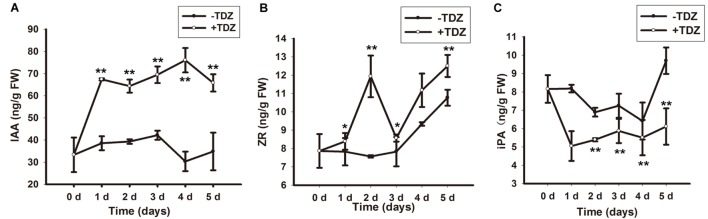
**Endogenous IAA, ZR and iPA levels in callus-rhizoids after culture on PIM.** Callus-rhizoids (0.2 g fresh) were collected after culture on PIM (1/2 MS + 20 mg/L TDZ) or 1/2 MS media without TDZ for 0, 1, 2, 3, 4, or 5 days. **(A)** The level of IAA and ZR was significantly increased by TDZ during the first 5 days of culture on PIM. **(B)** The endogenous level of ZR was repressed by TDZ. **(C)** The endogenous level of iPA was increased by TDZ, especially on day 2. Error bars represent the SE of three biological replicates. Asterisks indicate significant (^∗^*P* < 0.05) or highly significant (^∗∗^*P* < 0.01) differences calculated using the *t*-test (*n* = 9 for each treatment).

Auxin and CK determine regeneration in plant tissue culture. To clarify the effect of TDZ on endogenous levels of auxin and CK, we measured the concentration of IAA (endogenous auxin), iPA and ZR (both endogenous CKs). In callus-rhizoids, TDZ increased IAA and ZR levels (**Figures 1A,B**) and decreased the level of iPA (**Figure 1C**) during the first 5 days. The increase in IAA concentration was smoother than that of ZR and iPA and peaked on day 4 (**Figure 1A**). The level of ZR increased rapidly, peaking on day 2, dipping on day 3, then increasing gradually again on days 4 and 5 (**Figure 1B**). The content of iPA was lower than that of ZR and IAA and remained stable from day 2 to 5 (**Figure 1C**).

In brief, TDZ promoted the accumulation of endogenous IAA and ZR directly in callus-rhizoids. This is in agreement with previous results in which TDZ acted as a substitute for both auxin and CK during plant tissue culture (Visser et al., 1992; Casanova et al., 2004). Moreover, the level of IAA was a little lower and ZR was significantly higher on day 2 compared with days 1 and day 3.

### TDZ Acts Primarily as a CK during PLB Formation

**FIGURE 2:**
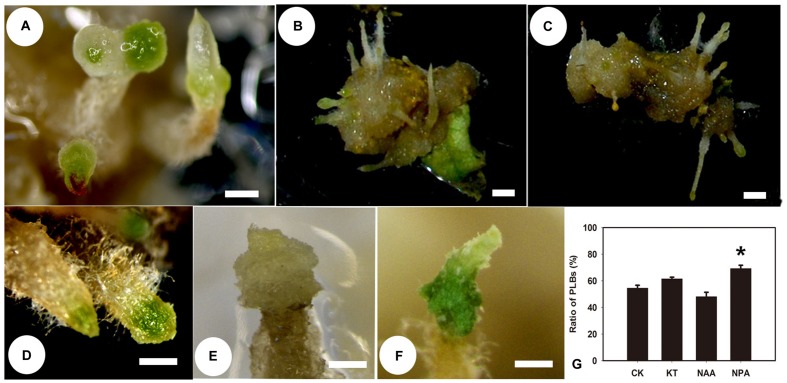
**Protocorm-like bodies (PLB) formation is affected by supplementary NAA, KT, and NPA.** Rhizoid tips began to swell and turn green after callus-rhizoids were transferred to PIM. This process was defined as PLB formation. The percentage of callus-rhizoids forming PLBs was calculated after culture on PIM for 9 days. **(A)** PLBs normally formed after 9 days of culture on PIM, and the rhizoid tip swelled and turned green. **(B,E)** PLB formation was inhibited after culture on PIM supplemented with 1.0 mg/L NAA, but some large white PLBs formed. **(C,D,F)** PLB formation increased when callus-rhizoids were cultured on PIM containing 0.1 mg/L KT. Rhizoid tips turned green earlier after KT treatment compared with the control. **(G)** Rate of PLB formation on medium with different PGRs compared with CK. Error bars represent the SE of three biological replicates. Asterisks indicate highly significant differences calculated using the *t*-test (^∗^*P* < 0.05) (*n* = 9–12 for each treatment).

Since the level of iPA was reduced in callus-rhizoids, we applied an additional treatment to callus-rhizoids cultured on PIM to investigate the effect of TDZ during the first 10 days of PLB formation. NAA or KT at 0.01, 0.1, or 1.0 mg/L was added to PIM, and then the number of PLBs on each callus-rhizoid was counted after culture for 9 days. The percentage of PLBs that formed decreased (**Figures 2B,G**; **Supplementary Table S4**) as NAA concentration increased and 1.0 mg/L NAA induced the fewest PLBs (only 48.12%) compared to the control (54.56%). In contrast, KT promoted PLB morphogenesis (**Figures 2C,G**; **Supplementary Table S4**) with 0.1 mg/L KT being the optimal concentration (PLBs forming in 61.54% of explants). However, 1.0 mg/L KT did not promote PLB formation, suggesting that this concentration was too high for PLB morphogenesis. In addition, PLBs were large and white on PIM containing 1.0 mg/L NAA (**Figures 2A,E**), but were small and green on PIM containing 1.0 mg/mL KT (**Figures 2D,F**). Rhizoid tips turned green 1 day earlier (they turned green on day 3) after KT treatment compared with the control (**Figures 2D,E**). Thus, we propose that TDZ acts in synergy with KT to promote PLB morphogenesis, but that PLB morphogenesis was inhibited by auxin. To test this hypothesis, we added 1 μM NPA, which is an inhibitor of auxin transport, to PIM, noting that the percentage of PLB formation increased to 69.23% (**Figure 2G**; **Supplementary Table S4**). Consequently, we concluded that TDZ mainly acted as a Z-type CK during the early stage of PLB morphogenesis.

### Expression of CK Signaling and Metabolism-Related Genes was Promoted by TDZ

**FIGURE 3:**
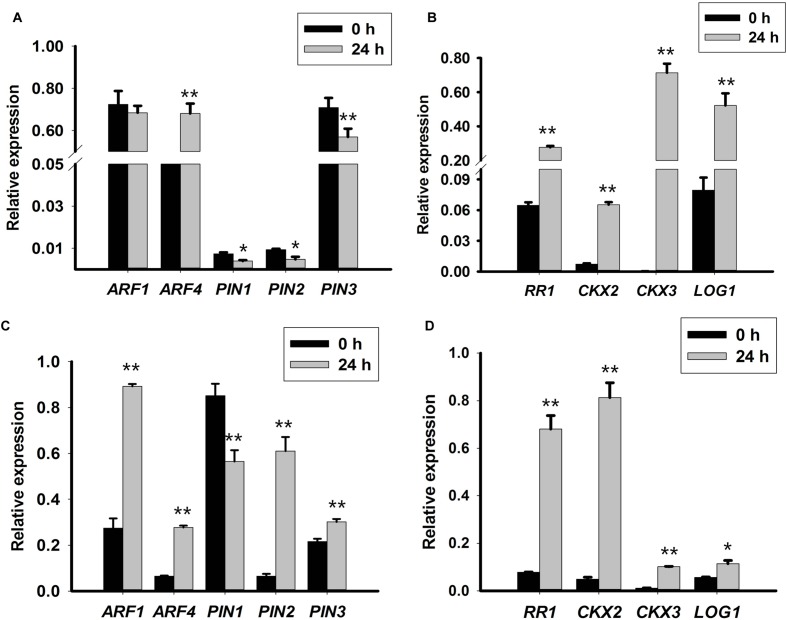
**Expression levels of auxin- and cytokinin-related genes in callus-rhizoids or rhizoid tips.** Callus-rhizoids (0.1–0.2 g fresh) or rhizoids only were collected after culture on PIM for 0 or 24 h. Quantitative real-time PCR was used to analyze genes expression levels. **(A,B)** Gene expression in callus-rhizoids. **(C,D)** Gene expression in rhizoid tips. Error bars represent the SE of three biological replicates. Error bars represent the SE of three biological replicates. Asterisks indicate significant (^∗^*P* < 0.05) or highly significant (^∗∗^*P* < 0.01) differences calculated using the *t*-test (*n* = 3 for each treatment).

The expression pattern of genes related to CK signaling and metabolism was evaluated to obtain insight into the function of TDZ during PLB formation. The expression of *RcRR1* increased in both callus-rhizoids and rhizoid tips (**Figures 3B,D**), which implied that CK signaling was promoted by TDZ. *RcLOG1* was used to analyze endogenous CK synthesis. Our results show that *RcLOG1* increased markedly in callus-rhizoids but the promoting effect of TDZ on *RcLOG1* transcription was weaker in rhizoid tips. These results indicate that TDZ promotes endogenous CK synthesis.

As mentioned above, the level of iP-type of CK (iPA) was reduced. Thus, the expression pattern of *RcCKX2* and *RcCKX3* was evaluated. The results show that both *RcCKX2* and *RcCKX3* expression increased both in callus-rhizoids and rhizoid tips (**Figures 3B,D**). However, *RcCKX2* transcription was more significantly promoted in rhizoid tips while transcription of *RcCKX3* was more significantly promoted in callus-rhizoids. This also confirmed our opinion that TDZ acted mainly as a CK during PLB morphogenesis.

Most importantly, we concluded that CK signaling, biosynthesis, and degradation were promoted during PLB formation by TDZ induction, and that genes related to CK signaling, biosynthesis and degradation participated in PLB development.

### Auxin Signaling Was Promoted but Auxin Transporters Were Repressed by TDZ

Transcription of *RcARF1* (**Figures 3A,C**) was repressed in callus-rhizoids but was slightly promoted in rhizoid tips. The transcription of *RcARF4*, which is a repressor of auxin signaling, was promoted (**Figures 3A,C**) both in callus-rhizoids and rhizoid tips. These results indicate that auxin signaling was mainly repressed through the promotion of *ARF* repressors even though the overall concentration of IAA increased in callus-rhizoids.

We assessed the transcription levels of *RcPIN1*, *RcPIN2*, and *RcPIN3* to clarify the role of auxin during PLB morphogenesis by TDZ induction. The results show that *RcPIN1* was repressed in both callus-rhizoids and rhizoid tips. Transcription of *RcPIN2* was inhibited in callus-rhizoids but was promoted in rhizoid tips. The expression level of both *RcPIN1* and *RcPIN2* was down-regulated in callus-rhizoids more than in rhizoid tips. Repression of *RcPIN1* expression may account for the accumulation of auxin (i.e., a decrease in its efflux) in callus-rhizoids (**Figures 3A,C**). The level of *RcPIN2* transcription was significantly promoted in rhizoid tips but that of *RcPIN1* was repressed. Transcription of *RcPIN3* was repressed in callus-rhizoids but promoted in rhizoid tips, indicating that *RcPIN3* expression was down-regulated by TDZ directly.

In summary, auxin transport was repressed in rhizoids by TDZ induction, and the balance of hormones in rhizoid tips was disturbed, promoting the conversion of cell fate.

### Expression of Root and Shoot Stem Cell-Related Genes Increased Following TDZ Induction

**FIGURE 4:**
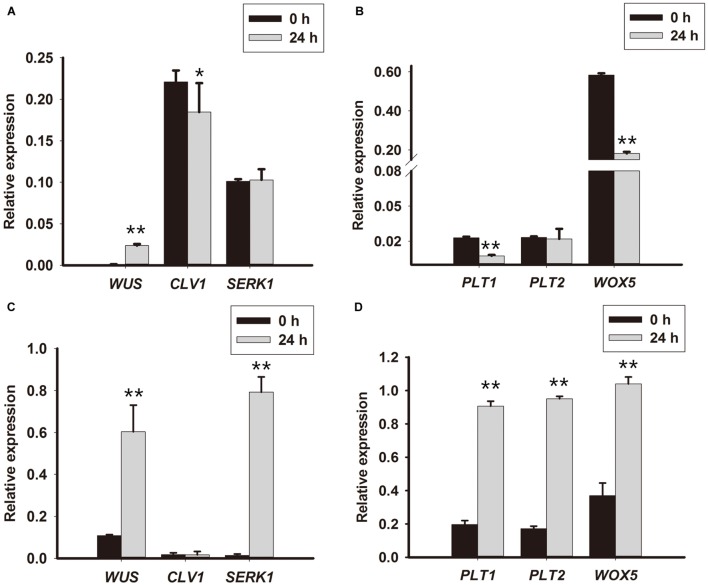
**Expression levels of root and shoot stem niche-related genes in rhizoid tips.** Callus-rhizoids (0.1–0.2 g fresh) and rhizoids were collected after culture on PIM for 0 or 24 h. Quantitative real-time PCR was used to analyze genes expression levels. **(A,B)** Gene expression in callus-rhizoids. **(C,D)** Gene expression in rhizoid tips. Error bars represent the SE of three biological replicates. Asterisks indicate significant (^∗^*P* < 0.05) or highly significant (^∗∗^*P* < 0.01) differences calculated using the *t*-test (*n* = 3 for each treatment).

To further understand how TDZ converted cell fate during PLB formation, root and shoot stem cell-related genes were analyzed in the first 10 days of PLB formation. The expression levels of *RcWUS*, *RcCLV1*, *RcPLT1*, *RcPLT2*, *RcWOX5*, *RcWUS*, and *RcSERK1* were evaluated to interpret the genetic mechanism underlying PLB morphogenesis. The results show that transcription of *RcPLT1* (**Figure 4B**) was repressed in callus-rhizoids but significantly promoted in rhizoid tips (**Figure 4D**). The expression of *RcPLT2* (**Figure 4B**) increased in callus-rhizoids and rhizoid tips (**Figure 4D**), but especially in rhizoids. *RcWOX5* expression (**Figure 4B**) decreased in callus-rhizoids but increased in rhizoids (**Figure 4B**). The expression of *RcSERK1* did not change in callus-rhizoids but increased significantly in rhizoid tips (**Supplementary Figure S2A**).

*RcWUS* transcription was promoted both in callus-rhizoids and rhizoids (**Figures 4A,C**), but *RcCLV1* expression was reduced in callus-rhizoids (**Figure 4C**) but only changed slightly in rhizoid tips (**Figure 4C**) after culture for 24 h. In addition, *RcWUS* expression increased in callus-rhizoids as TDZ concentration increased after culture for 16 h, with 20 mg/L TDZ being the optimal concentration to promote *RcWUS* transcription (**Supplementary Figure S1**). These results suggest that *RcWUS* could be induced directly by TDZ and that a feedback between *RcWUS* and *RcCLV1* had not been established within 24 h following TDZ induction.

The *RcWUS* gene is important for SAM and marks the position of shoot stem cell identity in plants. In our study, there was a significant increase in the expression of *RcWUS* in rhizoid tips, indicating that SAMs were established during the early stage of PLB morphogenesis. Consequently, we propose that TDZ triggered PLB formation following an increase in *RcWUS* expression.

### Cell Fate Was Converted to a Different Development Pathway in Rhizoid Tips

**FIGURE 5:**
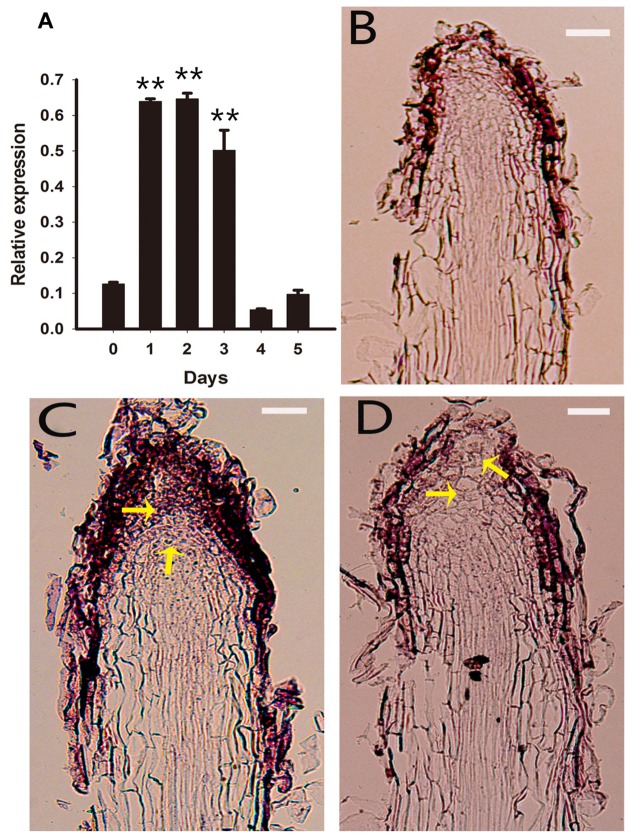
**Expression pattern of *RcWUS* in rhizoid tips.** Rhizoid tips (1 cm long) were collected after culture on PIM for 0, 1, 2, 3, 4, and 5 days. **(A)** Quantitative real-time PCR analysis of *RcWUS* expression pattern during the first 5 days of PLB formation. Error bars represent the SE of three biological replicates. Asterisks indicate significant differences calculated using the *t*-test (**P < 0.01) (*n* = 3 for each treatment). **(B–D)** *In situ* hybridization in longitudinal sections of rhizoid tips. *RcWUS* expression in rhizoid tips **(B)** before culture on PIM, **(C)** after culture for 3 days, or **(D)** after culture for 5 days. Bars = 2 μm.

To understand the developmental pathway during the early stage of PLB morphogenesis, we analyzed the expression of *RcSERK1*, *RcWUS* and *RcPLT1* in rhizoid tips using qRT-PCR and RNA *in situ* hybridization during the early stage of PLB formation. The results show that *RcWUS* expression increased significantly during the first 24–72 h (**Figure 5A**) then decreased on day 3. RNA *in situ* hybridization showed that *RcWUS* was expressed mainly in the outer layers (**Figure 5B**) of rhizoid tips before TDZ treatment (day 0). After treatment with TDZ for 3 days, *RcWUS* expression extended toward the epidermis, columella cells and even the QC (**Figure 5C**). However, on day 5, *RcWUS* expression decreased once again. The columella cells, which began to enlarge or convert into SAMS (**Figure 5D**), showed increased *RcWUS* expression on day 3.

In brief, our results show that the expression of *RcWUS* was transiently increased by TDZ during the early stage of PLB formation. *RcSERK1* expression increased significantly at 24 h (**Supplementary Figure S2A**) on PIM, corresponding to the *RcSERK1 in situ* hybridization results (**Supplementary Figures S2B–D**). These expression patterns of *RcSERK1* and *RcWUS* showed that cell fate converted to shoot stem cells and that developmental plasticity was induced in the RAM.

**FIGURE 6:**
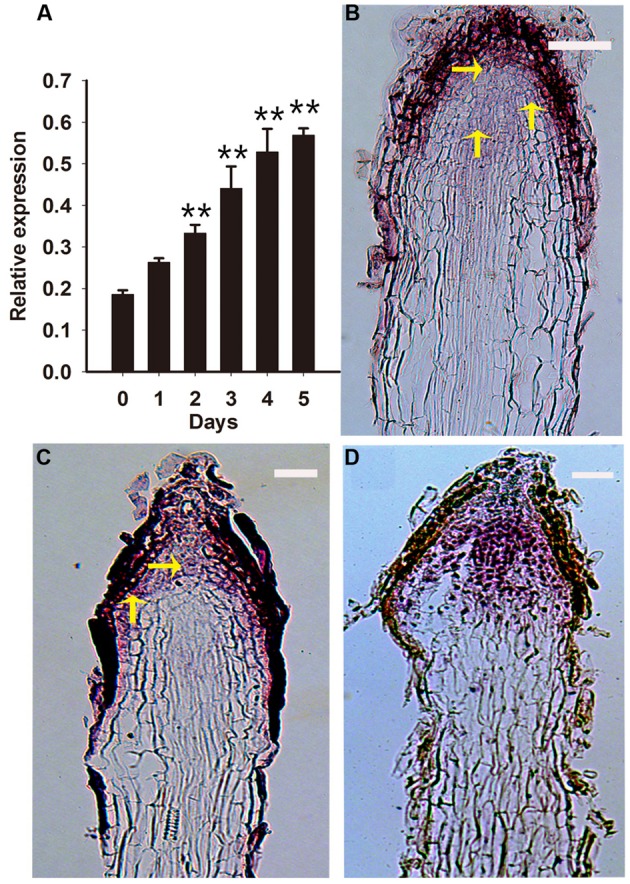
**Expression pattern of *RcPLT1* in rhizoid tips.** Rhizoid tips were collected after culture on PIM for 0, 1, 2, 3, 4, and 5 days. **(A)** Quantitative real-time PCR analysis of *RcPLT1* expression pattern during the first 5 days of PLB formation. Error bars represent the SE of three biological replicates. Asterisks indicate highly significant differences calculated using the *t* test (^∗∗^*P* < 0.01) (*n* = 3 for each treatment). **(B–D)** *In situ* hybridization in longitudinal sections of rhizoids. *RcPLT1* expression in rhizoid tips **(B)** before culture on PIM, **(C)** after culture for 3 days, or **(D)** after culture for 9 days. Yellow arrows show the position of *RcPLT1* expression. Bars = 2 μm.

*RcPLT1* is required for establishing competence to regenerate shoot progenitor cells in *Arabidopsis* (Kareem et al., 2015). Thus, we analyzed the expression pattern of *RcPLT1* to understand *de novo* shoot regeneration in PLB formation. Our results show that *RcPLT1* expression increased gradually from day 2 onward (**Figure 6A**). RNA *in situ* hybridization showed that the zone of *RcPLT1* expression extended toward the outer layer of the rhizoid around the stem cell niche (**Figures 6B,C**) where PLBs originated. In addition, *RcPLT1* expression increased significantly (**Figure 6D**) and extended to the whole rhizoid tip before swelling to a fully formed PLB. These results imply that a *de novo* shoot regeneration pathway may be activated and that cell fate was changed by TDZ induction.

### Sustainable Overexpression of *RcWUS* Repressed PLB Formation

**FIGURE 7:**
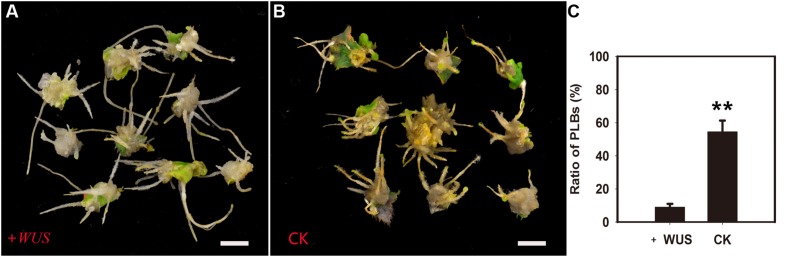
**Overexpression of *RcWUS* had little effect on PLB formation.** The leaves of *XVE::WUS* transgenic plants were used to produce callus-rhizoids according to Tian et al. (2008), which were then transferred to PIM that was supplemented or not with β-estradiol. **(A)** PLB formation was repressed by overexpression of *RcWUS* on PIM supplemented with 5 μM β-estradiol in transgenic line #8. **(B)** PLBs formed normally on PIM supplemented with 5 μM ethanol in transgenic line #8. **(C)** Percentage of PLB formation after culture in light for 9 days. Error bars represent the SE of three biological replicates. Asterisks indicate highly significant differences calculated using the *t*-test (^∗∗^*P* < 0.01) (*n* = 9–12 for each treatment).

*RcWUS* transcription was directly affected by TDZ (**Supplementary Figure S1**). In tobacco (*Nicotiana tabacum* L.), shoots can form from root tips following overexpression of the *WUS* gene by applying exogenous CKs (Rashid et al., 2007). Therefore, the *XVE::WUS* transgenic line was used to further explore the role of *RcWUS* during PLB formation. In this experiment, *RcWUS* transcription was controlled by the β-estradiol-inducible expression system in the pER8 vector. The callus-rhizoids that originated from the three transgenic lines (#5, #8, #9) were subcultured on PIM supplemented with or without 5 mM β-estradiol. PLB formation was slightly or significantly repressed (**Supplementary Table S3**) by an increase in *RcWUS* expression (**Figures 7A–C**). These results suggest that continuous expression of *RcWUS* repressed PLB formation and that *RcWUS* acted as a repressor during the early stage of PLB formation.

## Discussion

In *Rosa*, the process of PLB formation can be divided into three stages: early, bulking and shoot regeneration. The first 10 days are defined as the early stage in which cells of the rhizoid tip begin to divide and turn green. The bulking stage is defined as days 10–20 in which the rhizoid tip swells, the RAM disappears and multiple shoot meristem centers become established. From day 20 onward, leaf-like structures, pre-embryos, and secondary PLBs were visible, while shoot regeneration was complete after about 45 days (Liu et al., 2014). In the past several years, some research has focused on the genetic mechanisms underlying PLB formation in *R. canina*. *RcWUS* was isolated from *R. canina* PLBs and was ectopically expressed in tobacco resulting in shoot regeneration from *in vivo* leaves (Jiang et al., 2012). *RcSERK1* (Xu et al., 2011), *RcLEC1* (unpublished), *RcBBM*, and *RcABI3* (Yang et al., 2014a,b), which were identified from, and expressed exclusively in *R. canina* PLBs, may also be involved in PLB morphogenesis.

In the hypocotyl tissue of *Pelargonium* X *hortorum* Bailey, TDZ enhanced the accumulation and translocation of auxin (Murch and Saxena, 2001). In the tissue culture of lentil (*Lens culinaris* Medik.) cotyledonary nodes, TDZ at a concentration lower than 2.0 μM induced shoots buts at higher concentration (2.5–15 μM), somatic embryos formed. In plant tissue culture, TDZ exhibits CK activity in bioassays and is a highly stable compound. Previous studies have suggested that TDZ can modify the metabolism of endogenous CK (Capelle et al., 1983), or mimic an auxin response (Visser et al., 1992). Our results show that TDZ promoted the accumulation of IAA and a Z-type CK, ZR (**Figures 1A,B**) but reduced the level of an iP-type CK, iPA (**Figures 1C**) in callus-rhizoids of *R. canina*. KT induced PLB formation but NAA repressed it (**Figure 2G**; **Supplementary Table S4**). In addition, in callus-rhizoids, both auxin and CK responses were promoted by increased expression of *RcARF4* and *RcRR1* while auxin translocation was repressed by decreased expression of *RcPIN1*, *RcPIN2*, and *RcPIN3* (**Figures 3A,B**). Increased expression of *RcCKX2*, *RcCKX3* and *RcLOG1* in callus-rhizoids suggested that CK metabolism was altered by TDZ (**Figures 3C,D**). Moreover, application of NPA significantly promoted PLB formation (**Figure 2G**). Leaf-derived callus, which serves as an intermediary PLB-inducing tissue, subsequently forming rhizoids, responds directly to TDZ in PIM during PLB morphogenesis (**Figures 2B,C**). Consequently, these results suggest that TDZ acted mainly as a Z-type CK in the early stage (first 10 days) of PLB formation and that auxin transport was altered (**Figures 3A,C**).

Protocorm-like bodies originated from rhizoids tips and TDZ alone was able to trigger PLB morphogenesis. Cell fate of the RAM in rhizoid tips was converted and a SAM was established after triggering callus with TDZ. Thus, the effect of TDZ on rhizoid tips was different than that on callus-rhizoids. Our results showed that in rhizoid tips, both auxin and CK response were promoted by an increase in *RcARF1*, *RcARF4*, *RcRR1*, *RcPIN2* and *RcPIN3* expression (**Figures 3A,C,D**) but a decrease in the expression of the auxin transport protein gene *RcPIN1* (**Figure 3C**). CKs affect lateral root development by modulating auxin transport protein PIN1 (Rashotte et al., 2005; Marhavý et al., 2014). *RcRR1* was involved in rhizoid organogenesis in *R. canina* and primary root length and lateral root density increased and *AtPIN1* was repressed following over-expression of *RcRR1* in *Arabidopsis* (Gao et al., 2013). In the root elongation zone, the PIN1-dependent auxin transport stream flows into root tips through vascular tissue, and *PIN2*, *PIN3*, and *PIN7* mediate auxin flow out of root tips via epidermis cells, or ‘reflux’ of auxin basipetal transport (Friml et al., 2003; Blilou et al., 2005). Our results thus imply that auxin transport toward the RAM was inhibited by reducing the expression of *RcPIN1* in the vasculature whereas reverse transport was promoted by increasing the expression of *RcPIN2* and *RcPIN3* in epidermal cells. Furthermore, *RcRR1* maybe act as a repressor of *RcPIN1* transcription. These results also suggest that auxin efflux from rhizoid tips and the auxin gradient were altered by auxin transport in rhizoid tips, thereby disturbing the balance of auxin and CKs around stem cells, subsequently providing conditions that promoted the conversion of cell fate.

*CKX2*, *CKX3* and *LOG1* genes reportedly play an important role during SAM development in *Arabidopsis*, rice, soybean and tomato (Kurakawa et al., 2007; Yadav et al., 2009; Bartrina et al., 2011; Tamar et al., 2013; Wong et al., 2013). *AtCKX2* and *AtCKX3* were up-regulated by exogenous CKs (Werner et al., 2006), and transgenic *Pro35S:AtCKX2* and *Pro35S:AtCKX3 Arabidopsis* plant lines confirmed that endogenous CK is required during auxin-induced organogenesis *in vitro* (Pernisová et al., 2009). In *Arabidopsis*, *ckx3-ckx5* double mutants displayed increased *WUS* expression and larger SAMs (Bartrina et al., 2011). Expression of the *WUS* gene defines the organizing center of a SAM and WUS protein acts as a non-autonomous signal to maintain stem cells, at least in SAMs, and is sufficient to promote stem cell identity (Mayer et al., 1998; Yadav et al., 2010, 2013). In *Arabidopsis*, *WUS* expression has a complex interaction with CK signaling, biosynthesis, and degradation (Leibfried et al., 2005; Dello Ioio et al., 2008; Gordon et al., 2009; Yadav et al., 2009; Bartrina et al., 2011; Chickarmane et al., 2012). WUS represses type A-ARR inhibitors of CK signaling, thereby increasing CK signaling, which may in turn reinforce *WUS* expression (Leibfried et al., 2005). Dello Ioio et al. (2008) reported that the CK response factor *ARR1* activates transcription of the *Aux/IAA* gene *SHY2/IAA3* and that IAA3 protein in turns represses transcription of *PIN1*, resulting in a change of auxin distribution that promotes cell differentiation. In our results, the expression of *RcCKX2*, *RcCKX3* and *RcLOG1* increased in rhizoid tips, but that of *RcCKX2* was more significantly increased than in callus-rhizoids (**Figure 3**). *RcWUS* can respond to TDZ (**Supplementary Figure S1**) and was promoted both in callus-rhizoids and rhizoid tips (**Figures 4A,C**). However, *RcWUS* expression was repressed after a transient increase in expression in rhizoid tips (**Figure 5A**) and the location of *RcWUS* expression was limited to epidermal and columella cells (**Figures 5B,C,D**) before the bulking stage. An increase in *RcCKX2* and *RcCKX3* expression (**Figures 3B,D**) may have resulted in repressed expression of *RcWUS* and degradation of an iP-type CK, iPA. Together with the increased expression of *RcRR1*, we deduce that TDZ caused the increased in expression of *RcRR1*, *RcCKX2* and *RcCKX3* and that this increase in expression of *RcCKX2* and *RcCKX3* resulted in a decrease in *RcWUS* expression in epidermal and columella cells.

Ectopic WUS expression can result in shoot formation from the root tip region via a β-estradiol-inducible expression system in tobacco (Rashid et al., 2007). However, exogenous CK must be supplied for root tip bulking and shoot formation. The *SERK1* gene was used to indicate embryonic competence in *Medicago truncatula* and *Arabidopsis* (Hecht et al., 2001; Nolan et al., 2003). In our study, using three *RcWUS* transgene lines (#5, #8, #9), we found that sustained promotion of *RcWUS* transcription had no effect – or only a repressive effect – on PLB formation at an early stage (**Figures 7A–C**; **Supplementary Table S3**). The expression of *RcSERK1* showed that TDZ induced the embryonic competence of rhizoid tip cells (**Supplementary Figure S2**). In addition, *RcWUS* expression was significantly promoted from 24 to 72 h in rhizoid tips (**Figure 5A**), and the level of ZR was significantly higher on day 3 (**Figure 1B**) than earlier and later days. These results suggest that developmental events occurred in the callus-rhizoids or rhizoid tips within the first 5 days. *RcWUS* expression was transiently activated in epidermal and columella cells (**Figures 5B,C,D**), implying that SAM identity was established before the PLB bulking stage. This may be responsible for the formation of leaf-like structures or shoots directly on the epidermis of PLBs.

In *Rosa* PLBs, cell fate of the RAM in rhizoid tips is converted and a SAM becomes established within callus after being triggered by TDZ. *WOX5*, a marker of the root stem cell niche (Sarkar et al., 2007), maintains root fate with *PLT1*, *PLT2* (the mainly regulators of root stem cells) in *Arabidopsis* (Aida et al., 2004; Galinha et al., 2007). Previous research showed that shoots could be regenerated from multiple tissues through a lateral root developmental pathway in *Arabidopsis*, in which the root marker gene *WOX5* was activated during callus induction (Sugimoto et al., 2010; Ikeuchi et al., 2013). In our study, the expression of root stem cell genes *RcWOX5*, *RcPLT1* and *RcPLT2* was reduced in callus-rhizoids but increased significantly in rhizoid tips. Accompanying the bulking stage of PLBs, we deduced that the dedifferentiation of rhizoids was triggered via an intermediate callus pathway in the early stage of PLB formation. In *Arabidopsis*, the root stem cell regulator *PLT1* must be activated to establish the competence of shoot regeneration progenitor cells (Kareem et al., 2015). In our study, we showed that *RcPLT1* was activated in root stem cell niches (**Figure 6A**) and then expanded to the entire rhizoid tip (**Figures 6B–D**). Our results of the increased expression of root stem cell-related genes (*RcWOX5*, *RcPLT1*, and *RcPLT2*) (**Figures 4B,D** and **6**) suggest that the dedifferentiation pathway and *de novo* shoot regeneration program were activated in the center of rhizoid tips. *WOX5* reportedly represses the differentiation of columella cells in *Arabidopsis* (Sarkar et al., 2007), so an increase in *RcWOX5* expression in our study (**Figure 4D**) may be another cause for the decrease in *RcWUS* expression (**Figure 5A**) in epidermal and columella cells (**Figures 5B–D**). This is the first study to demonstrate the mechanism of conversion of a RAM to a SAM.

Our study has elaborated how TDZ triggers PLB morphogenesis via a change in the levels of endogenous auxin and CKs, and offers unique insight into the development pathway of columella cells and root stem cells during PLB formation in *Rosa*. The molecular mechanism of TDZ action in auxin and CK signaling, biosynthesis, and degradation was dissected, providing a better theoretical reference for using TDZ in practice, i.e., *in vitro* experiments in *Rosa* related to developmental studies. These hormones and developmental pathways intersect at multiple levels, and further studies are required to identify the direct regulator and stress signaling factors during plant regeneration *in vitro* induced by TDZ.

## Author Contributions

YK and LZ conceived and designed the research. CY and YK conducted the experiments. YK and CY performed the assays. QZ, GL, and JN prepared the plant material. YK, CC, and ZM took the photographs and analyzed the data. JT interpreted the experimental design and data. YK, CY, SJ, JT, and LZ co-wrote the manuscript. All the authors read and approved the manuscript.

## Conflict of Interest Statement

The authors declare that the research was conducted in the absence of any commercial or financial relationships that could be construed as a potential conflict of interest.
