# Supplementary material for: Thidiazuron Triggers Morphogenesis in Rosa canina L. Protocorm-Like Bodies by Changing Incipient Cell Fate
Source: Front Plant Sci. 2016 May 4;7:557. doi: 10.3389/fpls.2016.00557 (PMC4855734; doi:10.3389/fpls.2016.00557)
Supplement: Supplementary file 1 [file Table_1.DOCX]

**Thidiazuron Triggers Morphogenesis in *Rosa* *canina* L. Protocorm-like bodies by Changing Incipient Cell Fate**

Yaping Kou^1^, CunquanYuan^2^, Qingcui Zhao^1^, Guoqin Liu^1^, Jing Nie^1^, Zhimin Ma^1^, Chenxia Cheng^1^, Jaime A. Teixeira da Silva^3^, Liangjun Zhao^1*^

^1^Department of Ornamental Horticulture and Landscape Architecture, China Agricultural University, Beijing, China.

^2^National Engineering Research Center for Floriculture, Beijing Forestry University, Beijing

^3^P. O. Box 7, Miki-cho post office, Ikenobe 3011-2, Kagawa-ken, 761-0799, Japan

Corresponding author e-mail: [zhaolj5073@sina.com](mailto:zhaolj5073@sina.com)

Supplementary Table S1:

Primers for quantitative real-time PCR

| Gene name | Primer sequences (forward/reverse) |
| --- | --- |
| *RcARF1* | 5'-TACCTTGAAGCTCGCAGTCACA-3'  5'-CAACAATCGTGCCACTGAACCT-3' |
| *RcARF4* | 5'-ATGTCTGCTGCTCCCTCTTCTC-3'  5'-CTAACCAAGCAAGCCACAACCA-3' |
| *RcCKX2* | 5'-ACTCATGGCTCCTCTCCTCCT-3'  5'-GAACTGCGGCTGCGAATTGT -3' |
| *RcCKX3* | 5'-GTTTGGCGTGGCTGCGAAA-3'  5'-GCCGTGTTGTGGTAGGTGGT-3' |
| *RcCLV1* | 5'-GGAGGTGCTGCATGGGTCAAAG-3'  5'-ATCAGAGGCGAACAGTCGTGGT-3' |
| *RcLOG1* | 5'-AGGAACCATAATTGCCGGGTT-3'  5'-TGGACTGCTGAAGCTAGGGA-3' |
| *RcPLT1* | 5'-CCTGAACGCCGTCACCAACTTC-3'  5'-AAGGTGGAGCCCAGAGCAATCA-3' |
| *RcPLT2* | 5'-TGAACGCCGTCACCAACTTCG-3'  5'-AGCCTCTTTCAGCCGCTTTGC-3' |
| *RcPIN1* | 5'-TACTCCGAGACCTTCCAACTACG-3'  5'-TCCACCGCCACCACTTCC-3 |
| *RcPIN2* | 5'-GGCGAAGAAAGCAGGAAGA-3'  5'-GGTGGGTACGACGGAACA-3 |
| *RcPIN3* | 5'-ATGCCTCCGGCGAGCGTTAT-3'  5'-AGCGATGAGCGACCAGATGAGA-3' |
| *RcRR1* | 5'-CCACCTCCTCCCATTCCCATAG-3'  5'-ACTGAGAATCTGCGGCCATCC-3' |
| *RcSERK1* | 5'-GGCTATTGCTGGTGGAGTTGC-3'  5'-TCCTCCTCCGCAGGTACATCA-3' |
| *RcWUS* | 5'-AGCAACCAACCGAAGATGGA-3'  5'-GCTCTGCAGTTGGAGACCTAA-3' |
| *RcUBI2* | 5'-CTCCTTCACGGAATCCAACAC-3'  5'-AGTTGTCCCCTCAACTCAACCAGC-3' |
| *RcWOX5* | 5'-GGCGGTGGTGGTAATGGTGGTA-3'  5'-GGGTTCGGAGTCCAGACCTGAA-3' |
| *Rc18s* | 5'-CGCTACACTGATGTATTCAACGAGC-3'  5'-ACAATAATCCTTCCGCAGGTTCACC-3' |

Supplementary Table S2:

Primers for RNA probe

| Gene name | Primer sequence (forward/reverse) | Length (bp) |
| --- | --- | --- |
| *RcWUS* | 5'-TAATACGACTCACTATAGGG-3' | 268 |
|  | 5'-ATCTTCTCCTTGTTGTTCCA-3' |  |
| *RcPLT1* | 5'-GGCAGGCAAGGATAGGCAGAGT-3' | 520 |
|  | 5'-GGGCTTTGGCTTTGCTGGTGAA-3' |  |
| *RcSERK1* | 5'-AACGCCCACCATCCCAACCA-3' | 577 |
|  | 5'-GCCAAGCCATCACCTTCAAGCA-3' |  |

Supplementary Table S3:

Percentage of PLB formation in different *RcWUS* transgenic lines

| **Lines** | **PLB formation (%)** | |
| --- | --- | --- |
|  | **β-Estradiol** | **Ethanol** |
| 8# | 9.00 ± 0.5 | 54.56 ± 1.24 |
| 9# | 41.02 ± 1.14 | 51.52 ± 1.04 |
| 5# | 46.02 ± 1.08 | 49.52 ± 1.24 |

Means ± SE of three biological replicates are shown. Each replicate contains 25-45 explants. Significant differences were calculated by a *t*-test. β-Estradiol was used to increase the expression level of *RcWUS* in the *XVE::RcWUS* transgenic plant lines while ethanol was used to dissolve β-estradiol.

Supplementary Table S4:

Percentage of explants forming PLBs in the presence of difference concentrations of 1-naphthalene acetic acid (NAA) and kinetin (KT) supplied in PLB induction medium (PIM).

| Plant growth regulators in PIM | Conc. (mg/L) | Explants forming PLBs (%) |
| --- | --- | --- |
| KT | **0.01** | 55.19 ± 4.0 |
|  | **0.1** | 69.23 ± 1.5 |
|  | **1.0** | 50.99 ± 3.2 |
| NAA | **0.01** | 51.21 ± 2.6 |
|  | **0.1** | 49.89 ± 1.9 |
|  | **1.0** | 48.12 ± 3.5 |

Means ± SE of five biological replicates are shown. Each replicates contains 25-45 explants. Significant differences were calculated by a *t*-test.


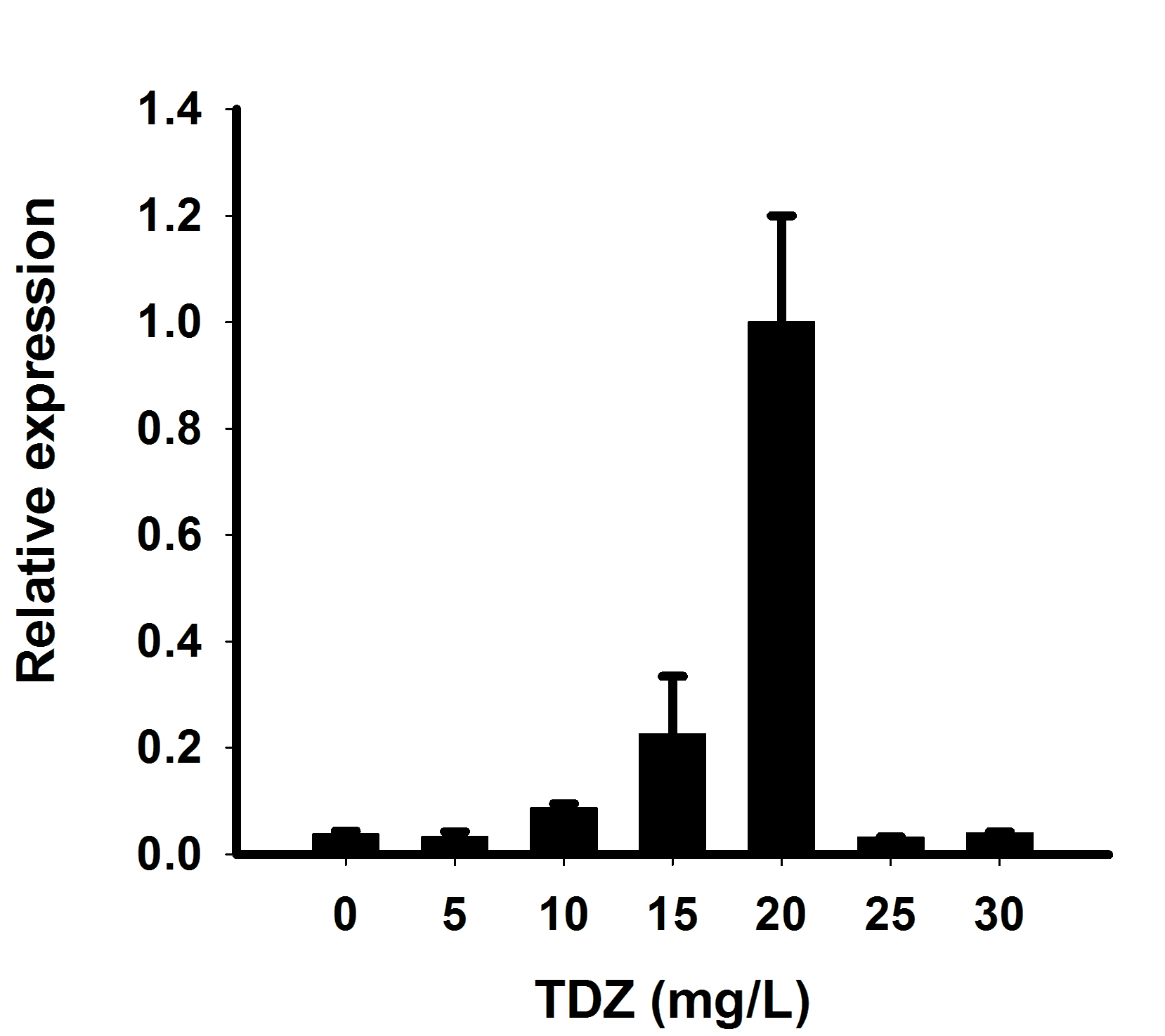


Supplementary Figure S1: *RcWUS* expression was regulated by thidiazuron (TDZ) in callus-rhizoids after culture in PLB induction medium (PIM) for 16 h.


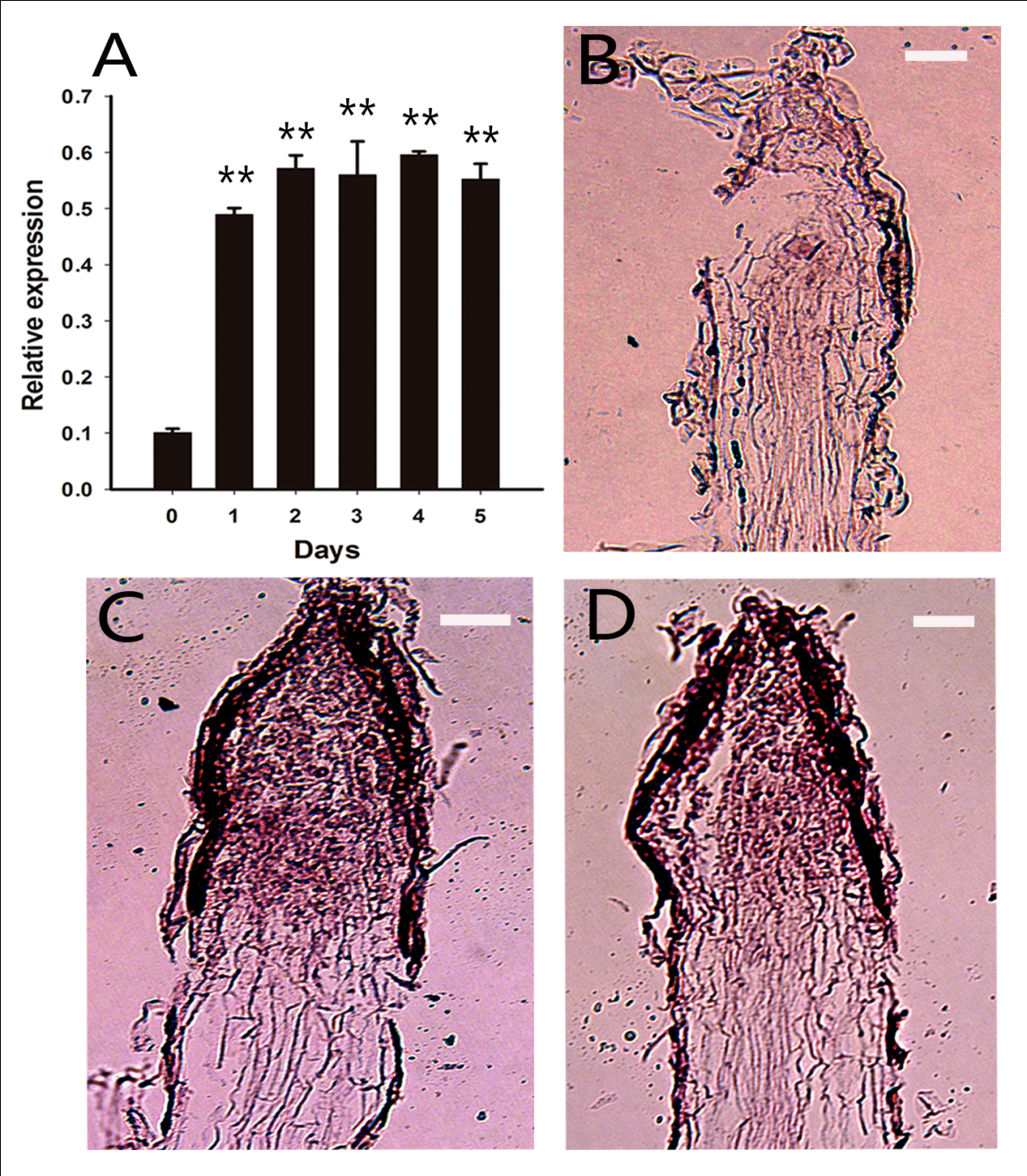


Supplementary Figure S2: Detection of expression pattern of *RcPSERK1* in rhizoid tips, which were collected after culture on PLB induction medium (PIM) for 0, 1, 2, 3, 4, and 5 days. (A) Quantitative real-time PCR analysis of *RcSERK1* expression pattern during the first 5 days of PLB formation. Error bars represent the SE of three biological replicates. Asterisks indicate significant differences calculated using the *t* test (***P*<0. 01). (B-D) *In situ* hybridization in longitudinal sections of rhizoids. *RcSERK1* expression in rhizoid tips (B) before culture on PIM, (C) after culture for 3 days, or (D) after culture for 5 days. Bars = 2 µm.
