# Supplementary figures and images for: Thidiazuron Triggers Morphogenesis in Rosa canina L. Protocorm-Like Bodies by Changing Incipient Cell Fate
Source: Front Plant Sci. 2016 May 4;7:557. doi: 10.3389/fpls.2016.00557 (PMC4855734; doi:10.3389/fpls.2016.00557)

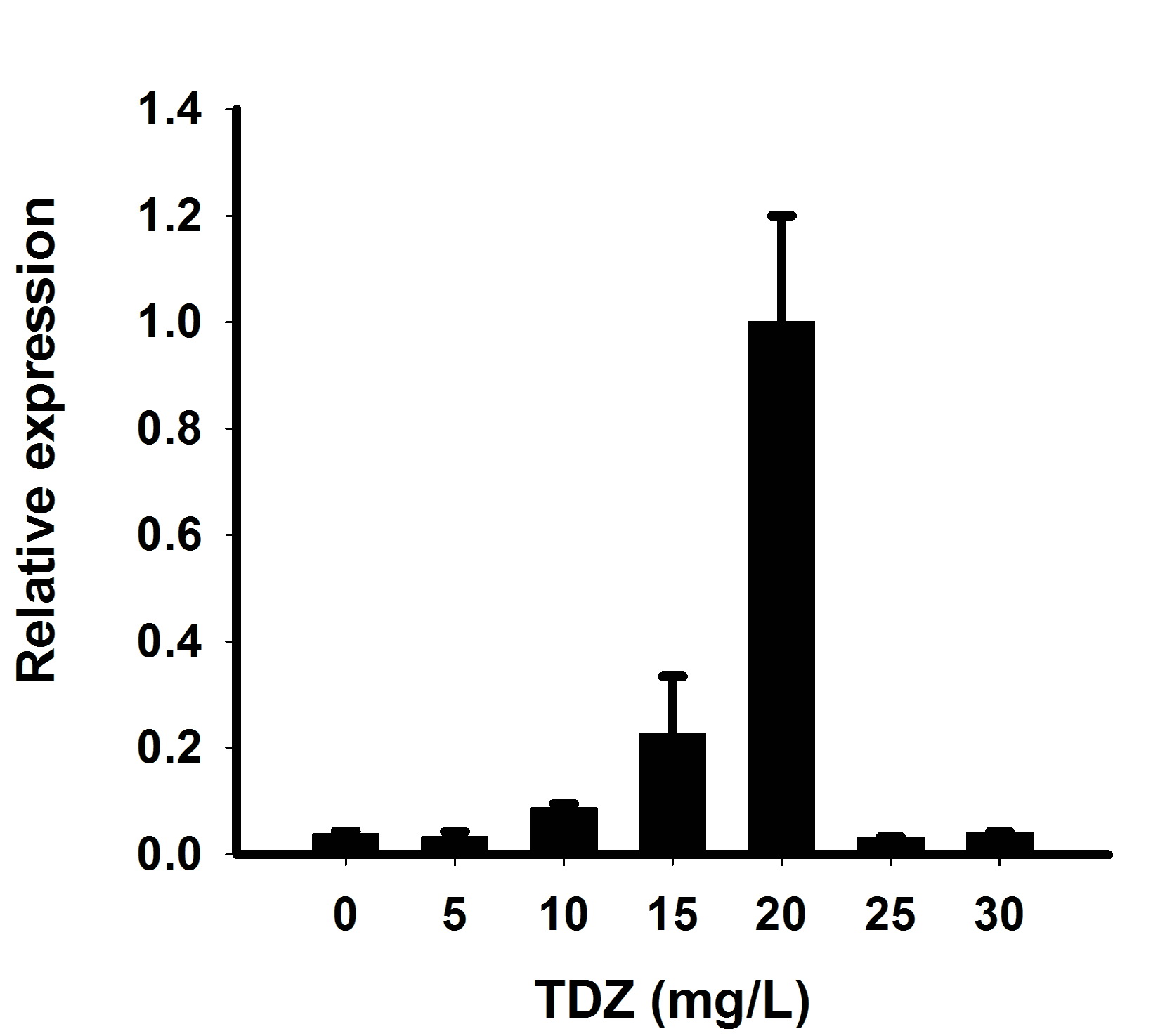

Supplement: Supplementary file 2 [file Image_1.JPEG]

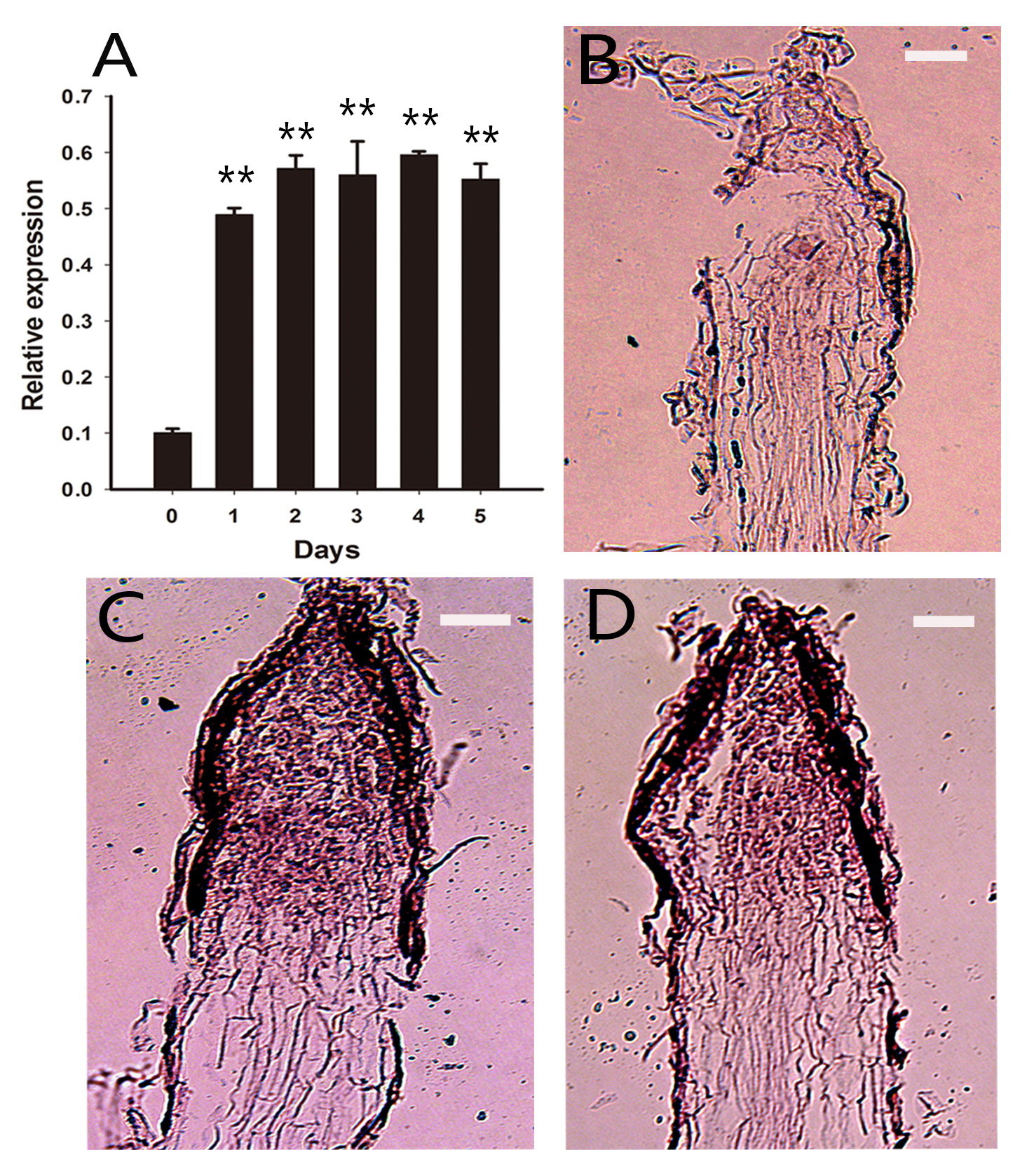

Supplement: Supplementary file 3 [file Image_2.JPEG]
